# Multi-Parametric Deep Learning Model for Prediction of Overall Survival after Postoperative Concurrent Chemoradiotherapy in Glioblastoma Patients

**DOI:** 10.3390/cancers12082284

**Published:** 2020-08-14

**Authors:** Han Gyul Yoon, Wonjoong Cheon, Sang Woon Jeong, Hye Seung Kim, Kyunga Kim, Heerim Nam, Youngyih Han, Do Hoon Lim

**Affiliations:** 1 Department of Radiation Oncology, Samsung Medical Center, Sungkyunkwan University School of Medicine, Seoul 06351, Korea; hangyul.yoon@samsung.com; 2 Samsung Advanced Institute for Health Science & Technology (SAIHST), Sungkyunkwan University School of Medicine, Seoul 06351, Korea; wonjoongcheon@ncc.re.kr (W.C.); sharkj@g.skku.edu (S.W.J.); 3 Proton Therapy Center, National Cancer Center, Goyang 10408, Korea; 4 Statistics and Data Center, Research Institute for Future Medicine, Samsung Medical Center, Seoul 06351, Korea; hyeseung.kim@sbri.co.kr (H.S.K.); kyunga.j.kim@samsung.com (K.K.); 5 Department of Radiation Oncology, Kangbuk Samsung Hospital, Sungkyunkwan University School of Medicine, Seoul 03181, Korea; heerim.nam@samsung.com

**Keywords:** glioblastoma, survival prediction, deep learning, radiomics

## Abstract

This study aimed to investigate the performance of a deep learning-based survival-prediction model, which predicts the overall survival (OS) time of glioblastoma patients who have received surgery followed by concurrent chemoradiotherapy (CCRT). The medical records of glioblastoma patients who had received surgery and CCRT between January 2011 and December 2017 were retrospectively reviewed. Based on our inclusion criteria, 118 patients were selected and semi-randomly allocated to training and test datasets (3:1 ratio, respectively). A convolutional neural network–based deep learning model was trained with magnetic resonance imaging (MRI) data and clinical profiles to predict OS. The MRI was reconstructed by using four pulse sequences (22 slices) and nine images were selected based on the longest slice of glioblastoma by a physician for each pulse sequence. The clinical profiles consist of personal, genetic, and treatment factors. The concordance index (C-index) and integrated area under the curve (iAUC) of the time-dependent area-under-the-curve curves of each model were calculated to evaluate the performance of the survival-prediction models. The model that incorporated clinical and radiomic features showed a higher C-index (0.768 (95% confidence interval (CI): 0.759, 0.776)) and iAUC (0.790 (95% CI: 0.783, 0.797)) than the model using clinical features alone (C-index = 0.693 (95% CI: 0.685, 0.701); iAUC = 0.723 (95% CI: 0.716, 0.731)) and the model using radiomic features alone (C-index = 0.590 (95% CI: 0.579, 0.600); iAUC = 0.614 (95% CI: 0.607, 0.621)). These improvements to the C-indexes and iAUCs were validated using the 1000-times bootstrapping method; all were statistically significant (*p* < 0.001). This study suggests the synergistic benefits of using both clinical and radiomic parameters. Furthermore, it indicates the potential of multi-parametric deep learning models for the survival prediction of glioblastoma patients.

## 1. Introduction

Glioblastoma is the most common primary malignant brain tumor in adults, which remains a fatal disease [1]. Even with surgery and postoperative concurrent chemoradiotherapy (CCRT), the five-year overall survival (OS) rate for glioblastoma is 9.8%, and the approximate median OS is only about 15 months [2,3]. However, the survival outcomes of glioblastoma are heterogeneous among patients and are known to be relevant to numerous clinical and genetic factors [4,5,6,7,8].

Recently, several studies have attempted to analyze this heterogeneity of prognosis in glioblastoma patients by using features from medical images [9]. The development and improvement of numerous computational algorithms (such as machine learning) have rendered these “radiomic” studies more feasible. Using machine learning, many recent studies have built prediction models that take radiomic features as inputs and predict several clinical variables for glioblastoma, including survival outcomes [10,11,12,13,14,15,16,17,18]. However, these studies have several limitations. Many studies used handcrafted features from a region of interest (ROI) delineated by humans or features selectively determined by researchers; as such, the reproducibility of these methodologies cannot be guaranteed [10,11,13,15,16]. This is mainly due to their use of machine learning algorithms that are not based on deep learning. In these studies, the feature extraction and prediction models are separated using conventional machine learning algorithms. Thus, researchers are required to either select features using evaluation methods or to redefine features using dimension-reduction techniques such as principal component analysis. In contrast, a deep learning model can automatically extract features and integrate the feature extraction and prediction processes into a single model. In addition, several studies did not include patients’ clinical information in their prediction model, using only image data as input parameters [15,16]. Considering that there have been already several studies where clinical metrics were married to radiological measures for the evaluation, this problem becomes more significant [19,20,21]. Furthermore, very few studies predicted survival outcomes as continuous—rather than categorical (long- or short-term)—variables in glioblastoma patients [10,12].

To summarize, although deep learning has several strengths in image processing (such as automated feature extraction), only a few studies have used deep learning frameworks to predict the survival outcomes of glioblastoma. Of these studies, models predicting survival outcomes as continuous variables were rarely used. Moreover, despite their impact on the prognosis of glioblastoma, significant clinical variables were not fully integrated into the radiomics models of previous studies. Therefore, in this study, we built neural network-based deep learning models that (1) do not require an ROI delineation and a pre-defined artificial feature extraction or manual selection process; (2) predict the survival outcomes of glioblastoma as OS by month, rather than with categorical variables; (3) use clinical features and radiomic data together; and (4) integrate feature extraction and an OS-prediction algorithm into one model. This study aimed to evaluate the predictive performances of the deep learning models and identify whether the addition of clinical features—such as personal, genetic, treatment, and radiomic factors—delivers synergistic benefits in survival predictions.

## 2. Results

### 2.1. Patient Characteristics

**Figure 1:**
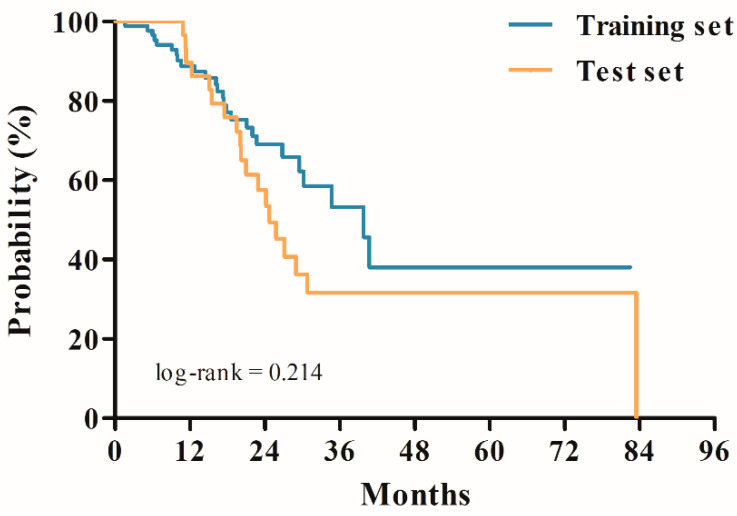
Kaplan–Meier survival curves for overall survival.

The patient characteristics are summarized in Table 1. No significant differences were observed between the training and test sets in terms of baseline characteristics such as age, sex, Eastern Cooperative Oncology Group (ECOG) performance status, resection status, isocitrate dehydrogenase (IDH) mutation, O-6-methylguanine-DNA-methyltransferase (MGMT) hypermethylation, and additional adjuvant temozolomide (TMZ) cycles after CCRT. Furthermore, no difference was observed in OS between the two groups in terms of survival analysis using the Kaplan–Meier estimate (*p*-value = 0.214) (Figure 1).

### 2.2. Model Performance Measured by C-Index and Integrated Area Under the Time-Dependent Receiver Operating Characteristic (ROC) Curve (iAUC)

**Figure 2:**
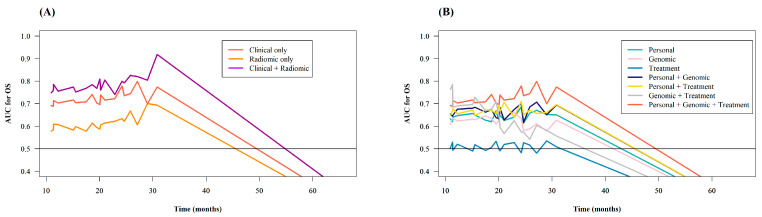
(**A**) and (**B**): Time-dependent receiver operating characteristic (ROC) curves of the models.

Table 2 lists the root mean squared error (RMSE), the square root of the mean squared residuals, and Pearson’s correlation coefficient between the ground truth and predicted OS in each model. M_CR_, the model which used both the clinical and radiomic features, showed the highest correlation coefficient (0.788) and the lowest RMSE (14.21 ± 23.07) among all the models in the study. However, RMSEs and correlation coefficients are not ideal evaluation metrics for survival-prediction models, mainly because of censored observations in survival data. Therefore, we calculated Harrell’s C-index and iAUC values of each model, and Table 3 lists the C-indexes and iAUC values of the models for OS prediction. Compared with the models using one type (M_C1a_, M_C1b_, M_C1c_) or two types (M_C2a_, M_C2b_, M_C2c_) of clinical features, the model using all three types of clinical features (M_C3_) showed a higher C-index (0.693 (95% confidence interval (CI): 0.685, 0.701)) and iAUC (0.723 (95% CI: 0.716, 0.731)) in most cases. Only the C-index of M_C2b_ (0.696 (95% CI: 0.688, 0.704)), which was slightly higher than that of M_C3_, was an exception. Furthermore, M_CR_ showed a higher C-index (0.768 (95% CI: 0.759, 0.776)) and iAUC (0.790 (95% CI: 0.783, 0.797)) than M_C3_ (C-index = 0.693 (95% CI: 0.685, 0.701); iAUC = 0.723 (95% CI: 0.716, 0.731)) and M_R_ (C-index = 0.590 (95% CI: 0.579, 0.600); iAUC = 0.614 (95% CI: 0.607, 0.621)). To summarize, M_CR_ had the highest predictive ability of all models in the study (Figure 2).

We statistically validated the synergistic effects of the clinical and radiomic features using 1000-times bootstrapping; the results are summarized in Table 4. The use of both clinical and radiomic features significantly improved the C-index compared with using the clinical (value difference = 0.074 (95% CI: 0.070, 0.078), *p* < 0.001) or the radiomic (value difference = 0.178 (95% CI: 0.174, 0.183), *p* < 0.001) features alone. The iAUC of the model using both features was also significantly higher than that of the model using clinical features alone (value difference = 0.067 (95% CI: 0.064, 0.070), *p* < 0.001) and the model using radiomic features alone (value difference = 0.176 (95% CI: 0.174, 0.179), *p* < 0.001).

## 3. Discussion

This study investigated the predictive performance of multiparametric deep learning models for OS prediction in glioblastoma patients. The deep learning model employing both clinical and radiomic features showed a higher C-index and iAUC than any other model in the study. Furthermore, the benefits of utilizing both features rather than clinical or radiomic features alone were also evaluated using the bootstrapping method. The improvement in C-index and iAUC achieved by combining the two features was found to be statistically significant.

Several studies have designed radiomics models to predict the survival outcomes of glioblastoma [10,11,12,13,15,16]. Lao et al. [11] built a radiomics model with handcrafted and deep features from a dataset describing 75 patients. Compared with the Cox regression models of traditional risk factors (such as age and Karnofsky performance score [22]), the radiomics model showed improved performance in terms of OS prediction and the stratification of patients into low- and high-risk groups for a validation set containing 37 patients. Another study—also using a multiparametric radiomics model—demonstrated the predictive superiority of the radiomics model over models using conventional clinical factors for OS prediction [23]. Moreover, in the study of Bae et al. [10], radiomics models based on a random survival forest algorithm were used to predict the OS and progression-free survival of glioblastoma patients. A model incorporating radiomic features from the ROI—using clinical and genomic features—showed better predictive power than the models using each feature type alone.

However, as mentioned in the introduction, most previous studies use machine learning algorithms that are not based on deep learning. This has necessitated handcrafted features from the ROI or manual feature selection by clinicians, which is a major drawback of non-deep learning machine learning models. In contrast, this study used a model structure based on a convolutional neural network (CNN), a deep learning algorithm that has repeatedly shown promising ability to process and analyze image data [24,25,26,27,28]. Alongside its strengths in image analysis, CNN also presents other advantages such as automated feature extraction and selection. Owing to this benefit of CNNs, ROI delineation and artificial feature-selection processes could be omitted in this study. This characteristic of CNN and the fact that all MRI images in this study were obtained on the same machine may contribute to the reproducibility of our study results. Moreover, since it is relatively straightforward to adapt the network structure of models when using deep learning, we generated multiple prediction models with various combinations of input parameters. Thus, it became possible to identify the synergistic benefits obtained via the integrated use of clinical and radiomic features in this study.

CNN is a shift- or space-invariant deep learning model, which can automatically extract optimal features by itself from the given data to achieve the best performance. Thus, it is suitable for OS prediction considering positional relationship of features between MRI series [29]. In this study, we calculated the mean weight from the optimized weight of one-by-one convolution filter to analyze the weight of four pulse sequence MRI series. The one-by-one convolution network generated a weighted image (512 × 512 × 1) from the input image (512 × 512 × 36). As a result, MRI images of T1-weighted and apparent diffusion coefficient (ADC) images showed higher weight than T1-weighted images with contrast-enhancement (CE), or T2-fluid-attenuated inversion recovery (FLAIR) pulse sequence images (Figure S1).

To the best of our knowledge, no studies except one have built deep learning-based models to predict OS as a continuous variable, using both clinical and radiomic data together [12]. In the exceptional study just referred to, the CNN-based model using image data and clinical/genomic features showed a lower RMSE and higher correlation coefficient (177.0 ± 130.0 and 0.4695, respectively) than the random survival forest-based model (225.0 ± 136.0 and 0.1151, respectively) or the CNN-based model using magnetic resonance imaging (MRI) data only (261.0 ± 175.0 and 0.0587, respectively). However, owing to censored observations in survival data, RMSEs and correlation coefficients are not ideal evaluation metrics for survival-prediction models. Moreover, several critical clinical factors known to be significantly associated with the survival outcomes of glioblastoma (such as performance status and resection margin status) were not used in the study [4,5,30,31]. In contrast, our study calculated the C-index and iAUC values, which are more appropriate metrics for evaluating the predictive performance of survival-prediction models than the RMSE or correlation coefficient. The calculation of C-index and iAUC reflected individual survival status at each time point, which is not available in several statistical methods such as the standard ROC curve analysis [32,33]. Furthermore, our study included various clinical features known to be significantly associated with the survival outcomes of glioblastoma, which may have contributed to our more comprehensive analysis. Therefore, results from our study can overcome the limitations of the aforementioned study, and can address the clinical significance of multi-feature deep learning-based models in glioblastoma treatment.

The present study has several limitations. First, we retrospectively collected data from a single institution. Second, because our institution began to routinely acquire IDH mutation information in recent years, we excluded many patients due to a lack of genetic factors. Owing to this exclusion, we had to perform the study with a relatively small sample size. Additional studies with a large number of patients and a multi-center design are needed for the external validation of our results. Third, instead of Karnofsky performance score [22], which might have been a more idealistic criterion for evaluating patient’s performance, our study used ECOG performance status. Fourth, only the limited number of patients received lower dose CCRT, and none of the included patients received tumor-treating fields (TTFs) in this study. Considering the modest sample size of the study, this might have contributed to the deterioration of our models’ predictive performance. Fifth, the center images of each patient’s MRI data (which included the largest proportion of suspicious lesions), were selected by clinicians in this study. However, this limitation might be overcome in future studies by using auto-segmentation algorithms such as U-net [34,35]. Sixth, several radiomic features including relative cerebral blood volume (rCBV) were not incorporated into our prediction models. With consideration of the recent advances, feature extraction reflecting the relationship of rCBV or the geographical relationship of radiographic features between different MRI series can be performed in future studies [36]. Finally, our study only used pre-operative MRI images as the radiomic features and predicted OS. Although this disadvantage could have been mitigated by using post-operative clinical features, further studies are required to validate the clinical utility of CNN-based models on post-operative images.

## 4. Materials and Methods

### 4.1. Patient Selection

The medical records of patients with glioblastoma were retrospectively reviewed. These patients had all received surgery followed by CCRT with TMZ between January 2011 and December 2017 at the Samsung Medical Center (Seoul, Korea). This study was approved by our Institutional Review Board (IRB #2019-02-070) and was performed under the guidelines of the Declaration of Helsinki. The inclusion criteria were as follows: (1) availability of pre-operative MRI data, including T1-weighted images, T1-weighted images with contrast-enhancement (CE), T2-fluid-attenuated inversion recovery (FLAIR) images, diffusion-weighted imaging (DWI), and apparent diffusion coefficient (ADC) images; (2) completion of planned CCRT; and (3) availability of genetic profiles. Finally, 118 patients who met the inclusion criteria were included in the study. None of the patients received TTFs.

We obtained 14 clinical features of the included patients, and these features were classified into three categories: (1) “personal” factors (*n* = 3)—age, sex, and ECOG performance status; (2) “genetic” factors (*n* = 2)—IDH mutation status and MGMT hypermethylation status; (3) “treatment” factors (*n* = 9)—resection status, adjuvant TMZ cycles after CCRT (six cycles is the gold standard, according to the Stupp protocol [2] for glioblastoma), type of radiotherapy (RT), gross tumor volume (GTV), clinical target volume (CTV), and RT dose for the first RT, as well as GTV, CTV, and RT dose for the second RT planning (a boost RT plan after the first RT). GTV and CTV were used as continuous variables. Thereafter, patient data were allocated to the training and test datasets (in about 3:1 ratio, respectively) using the “createDataPartition” function from the “caret” R package. To avoid a significant difference in IDH mutation status between the two groups, patient data were allocated to the training and test datasets while maintaining the ratio of IDH-mutants of each dataset. No other variables were used for the stratification of the datasets.

### 4.2. Image Acquisition and Pre-Processing

All MRI images used in this study were taken at a single institution using identical 3.0 T MRI machines (Achieva, Philips Healthcare, Best, the Netherlands). The MRI images were reconstructed with matrix size of 512 × 512 × 22 by using four pulse sequences. Four sequences of preoperative MRI data were used as “radiomic” features, including (1) T1-weighted images, (2) T1-weighted images with CE, (3) T2-FLAIR images, and (4) ADC images. Repetition times/echo times were 500.0/10.0 ms and 11,000.0/125.0 ms in the T1 and FLAIR images, respectively. These images shared the same acquisition parameters, with a section thickness of 5.0 mm and a reconstructed axial image of a 512 × 512 matrix with a pixel spacing of 0.469 mm × 0.469 mm. T1-CE images were captured 5 min after injecting a gadolinium-based agent. Moreover, DWI was performed with the following parameters: *b*-values = 1000 and 0 s/mm^2^, repetition/echo times = 3000.0/82.0 ms, and section thickness = 5.0 mm. We used a 256 × 256 matrix with a pixel spacing of 0.9375 mm × 0.9375 mm. Based on the DWI of two different *b*-values, ADC images were acquired using an EWS Workstation (Philips Healthcare, Amsterdam, Netherlands).

The preoperative MRI images used in this study were resized to a width and height of 256 × 256 pixels, to fix the size of the input for the deep learning models. Considering the memory capacity of the graphics processing unit (GPU), a total of nine images were selected, based on the longest slice of glioblastoma from one sequence of data (± four slices). As a result, a total of 36 axial images (4 sequences × 9 images) from the four different image series were incorporated into our prediction model. The z-score normalization method was applied to the training data—including clinical and radiomic features—and the z-score parameters (mean and standard deviation) were determined. Then, the z-score parameters were independently applied to the test data.

### 4.3. Building Neural Network-Based Survival-Prediction Models

All procedures for building the deep learning-based OS-prediction models were performed using Google Tensorflow library version 1.14.0 (http://tersorflow.org) with an Nvidia GTX 1080Ti GPU. The OS prediction models were designed using clinical, radiomic, and both clinical and radiomic features. A total of nine neural network-based models were built in this study. These can be classified into five categories: (1) models with one type of clinical feature (M_C1a,_ M_C1b_, and M_C1c_), (2) models with two types of clinical feature (M_C2a,_ M_C2b_, and M_C2c_), (3) a model with all three types of clinical feature (M_C3_), (4) a model using radiomic features (M_R_), and (5) a model with both clinical and radiomic features (M_CR_). The clinical features used in each M_C_ were as follows: M_C1a_—personal only; M_C1b_—genomic only; M_C1c_—treatment only; M_C2a_—personal and genomic; M_C2b_—personal and treatment; M_C2c_—genomic and treatment; M_C3_—personal, genomic, and treatment.

**Figure 3:**
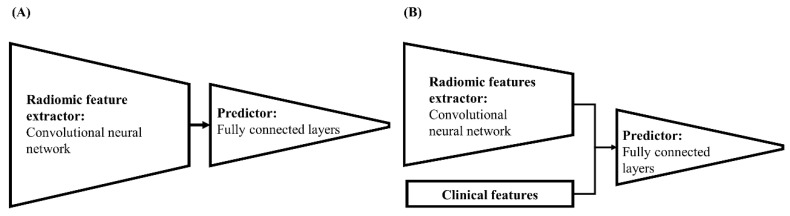
Diagrams of (**A**) the model using radiomic features and (**B**) the model using both clinical and radiomic features.

Each model consisted of an input layer, a hidden layer, and an output layer. The model details are as follows: (1) in the models using clinical features alone (M_C_), the size of the input layer was determined according to each model’s input variable numbers. Thereafter, the input data passed through a hidden layer composed of four fully connected layers (256, 128, 64, and 32 nodes, respectively) and a single output node to predict the OS. (2) In the case of the radiomic model (M_R,_ Figure 3A), 36 images from the four MRI sequences (shaped 256 × 256 × 36) were entered into the input layer of M_R_. The hidden layer of M_R_ was based on a CNN composed of seven convolution layers and four fully connected layers. The CNN of the hidden layer performed two functions. The one-by-one convolution filter in the first convolution layer determined the importance of weight for each of the 36 images. The remaining six convolution layers (shaped from 28 × 28 to 7 × 7) automatically extracted the radiomic features (Radiomic Feature Extractor). Then, the 256 extracted radiomic features were inputted to the fully connected layer (Predictor) to predict the OS. (3) The prediction model employing both the clinical and radiomic features (M_CR_, Figure 3B) had a similar CNN structure to M_R_, except for a single additional fully connected layer containing 242 nodes. The 36 images from the four MRI sequences were entered into the input layer and passed through the CNN in a manner similar to that of M_R_, and the Radiomic Feature Extractor in M_CR_ extracted 242 features using the additional fully connected layer. The 242 extracted radiomic features and 14 clinical features were then inputted to four fully connected layers to predict the OS. The architecture specifications of M_CR_ are listed in Table 5.

The batch size was empirically determined as 15; dropout and online augmentation techniques were applied to achieve generalizable performance. The online augmentation technique generated new images every epoch by using randomly selected parameters until the training procedure is over. The parameters of scaling, translation, rotation, and shear ranged from 95% to 100%, −3 to 3 pixels, −10 to 10 degrees, −3 to 3 degrees, respectively. The probability of image flip (left-right) was 50%. To manage the non-linearity, a leaky rectified linear unit activation function (LReLu) was located behind each layer [37]. After the input features had been passed from the input to the output layer, the loss was calculated (forward-propagation). The loss functions of the models were determined as the root mean squared error between the actual (ground-truth) and predicted OS; they were measured in months. In the back-propagation process, the hidden layer was optimized to minimize the loss using the adaptive moment estimation optimizer, with a learning rate of 0.0001. The training process was terminated when the loss became saturated on the smallest value, with a maximum of 5000 epochs. After the training was completed, the model structure and optimized parameters were saved, to be independently applied to the test set. The model performances were validated via 10-fold cross-validation.

### 4.4. Statistical Analysis

The primary objective of this study was OS prediction. The OS duration was calculated from the date of surgery to the date of the last follow-up (or death). Survival rates were calculated using the Kaplan-Meier method and were compared by log-rank tests. For a comparison of variables between groups, the Student’s *t*-test was employed for continuous variables, and the Chi-square test or Fisher’s exact test was used for categorical variables.

To evaluate the predictive performance of each model, Harrell’s C-index and the iAUC were calculated. The iAUC is defined as the weighted mean of the area-under-the-curve over a follow-up period, and a higher iAUC suggests the superior predictive performance of the model [38,39]. The C-index and iAUC were calculated using the “survival” R package, and the “risksetAUC” function from the “risksetROC” R package, respectively. To calculate differences in the C-index and iAUC between two models, the 95% CIs of each value were computed using 1000-times bootstrapping. The difference was considered statistically significant if 95% CI of the difference did not include a zero. In each comparison, we used Bonferroni correction to compensate for multiple comparisons. In addition, we calculated the RMSE, the square root of the mean squared residuals, and Pearson’s correlation coefficient between the ground truth and predicted OS in each model. All statistical analyses in this study were performed using the SAS software (version 9.4; Cary, NC, USA), and R (version 3.6.1; R Development Core Team, Vienna, Austria, http://www.r-project.org).

## 5. Conclusions

In conclusion, in this study, we proposed deep learning models for the prediction of OS in glioblastoma. In contrast to most previous studies, we predicted survival outcome as a continuous variable and automated the feature-extraction and selection processes by using CNN-based algorithms. Our findings suggest synergistic benefits of clinical and radiomic features; furthermore, they suggest future research directions in the building of multi-parametric deep learning models for the survival prediction of glioblastoma patients.

## Figures and Tables

**Table 1:** Baseline patient characteristics (*n* = 118).

Variables	Training Set (*n* = 88) ^a^	Test Set (*n* = 30)	*p*-Value
Age (years)	Median 59 (IQR 50.75–64.25)	Median 54.5 (IQR 48–65.5)	0.410
Survival time (months) ^b^	Median 17.60 (IQR 10.35–26.68)	Median 23.00 (IQR 17.03–33.59)	0.214 ^c^
Sex			0.531
Male Female	44 (50.0%) 44 (50.0%)	13 (43.3%) 17 (56.7%)	
ECOG Performance Status			0.753
0–1 2	73 (83.0%) 15 (17.0%)	27 (90.0%) 3 (10.0%)	
Resection			0.931
Gross total resection Subtotal resection	36 (40.9%) 52 (59.1%)	12 (40.0%) 18 (60.0%)	
IDH mutation			0.468
Yes No	7 (8.0%) 81 (92.0%)	4 (13.3%) 26 (86.7%)	
MGMT hypermethylation			0.921
Yes No	42 (47.7%) 46 (52.3%)	14 (46.7%) 16 (53.3%)	
Adjuvant TMZ cycles	Median 6 (IQR 4–6)	Median 6 (IQR 4.5–6)	0.300
Total radiotherapy dose			0.778 ^d^
≥60 Gy <60 Gy	73 (83.0%) 15 (17.0%)	26 (86.7%) 4 (13.3%)	

ECOG, Eastern Cooperative Oncology Group; IDH, isocitrate dehydrogenase; MGMT, O-6-methylguanine-DNA-methyltransferase; TMZ, temozolomide; IQR, interquartile range. ^a^ Using the “createDataPartition” function from the “caret” R package, patient data were allocated to the training and test datasets while maintaining the ratio of IDH-mutants of each dataset. ^b^ Described as mean ± standard deviation. ^c^ Using Kaplan–Meier analysis. ^d^ Using Fisher’s exact test.

**Table 2:** Root mean squared error and correlation coefficient of each model for overall survival prediction.

Model	Included Features	RMSE (Months) ^a^	Correlation Coefficient
M_C1a_	Personal only	16.96 ± 23.89	0.562
M_C1b_	Genomic only	19.88 ± 30.40	0.194
M_C1c_	Treatment only	25.18 ± 36.89	0.073
M_C2a_	Personal + Genomic	17.19 ± 22.96	0.579
M_C2b_	Personal + Treatment	16.64 ± 28.92	0.593
M_C2c_	Genomic + Treatment	29.18 ± 38.57	−0.222
M_C3_	Personal + Genomic + Treatment = Clinical	16.01 ± 26.54	0.712
M_R_	Radiomic only	17.14 ± 25.47	0.499
M_CR_	Clinical + Radiomic	14.21 ± 23.07	0.788

RMSE, root mean squared error. ^a^ Described as mean ± standard deviation.

**Table 3:** Harrell’s concordance index (C-Index) and integrated area under the time-dependent receiver operating characteristic curve (iAUC) values of each model for overall survival prediction.

Model	Included Features	C-Index (95% CI)	iAUC (95% CI)
M_C1a_	Personal only	0.644 (0.635, 0.653)	0.644 (0.636, 0.653)
M_C1b_	Genomic only	0.664 (0.656, 0.671)	0.641 (0.634, 0.649)
M_C1c_	Treatment only	0.562 (0.553, 0.570)	0.579 (0.572, 0.586)
M_C2a_	Personal + Genomic	0.696 (0.688, 0.704)	0.675 (0.666, 0.684)
M_C2b_	Personal + Treatment	0.665 (0.655, 0.675)	0.671 (0.663, 0.679)
M_C2c_	Genomic + Treatment	0.640 (0.630, 0.650)	0.664 (0.657, 0.672)
M_C3_	Personal + Genomic + Treatment = Clinical	0.693 (0.685, 0.701)	0.723 (0.716, 0.731)
M_R_	Radiomic only	0.590 (0.579, 0.600)	0.614 (0.607, 0.621)
M_CR_	Clinical + Radiomic	0.768 (0.759, 0.776)	0.790 (0.783, 0.797)

iAUC, integrated area under the time-dependent receiver operating characteristic curve; CI, confidence interval.

**Table 4:** Comparison of prediction performances measured using Harrell’s C-Index and iAUC value.

Index	Model 1	Model 2	Value Difference (95% CI) ^a^	*p*-Value ^b^
C-Index	Clinical only	Clinical + Radiomic	0.074 (0.070, 0.078)	<0.001
	Radiomic only	Clinical + Radiomic	0.178 (0.174, 0.183)	<0.001
iAUC	Clinical only	Clinical + Radiomic	0.067 (0.064, 0.070)	<0.001
	Radiomic only	Clinical + Radiomic	0.176 (0.174, 0.179)	<0.001

iAUC, integrated area under the time-dependent receiver operating characteristic curve; CI, confidence interval. ^a^ The value difference was calculated as (value of Model 2)−(value of Model 1) with 1000-times bootstrapping. ^b^ Using Bonferroni correction, the level of statistical significance in each comparison was set at 0.05/2 = 0.025.

**Table 5:** Layers and parameters of the prediction model employing both the clinical and radiomic features (M_CR_).

Layer		Filter Shape	Shape	Activation/Pooling
Input layer	Input layer	-	1 × 256 × 256 × 36 ^†^	None
Hidden layer: Extractor	Convolution layer	1 × 1 × 36 × 1	1 × 256 × 256 × 1	None/Max pooling
	Convolution layer	1 × 28 × 28 × 1	1 ×128 × 128 × 1	LReLu */Max pooling
	Convolution layer	1 × 14 × 14 × 1	1 × 64 × 64 × 1	LReLu/Max pooling
	Convolution layer	1 × 14 × 14 × 1	1 × 32 × 32 × 1	LReLu/Max pooling
	Convolution layer	1 × 7 × 7 × 1	1 × 16 × 16 × 1	LReLu/None
	Convolution layer	1 × 7 × 7 × 1	1 × 16 × 16 × 1	LReLu/None
	Flatten		1 × 1 × 256	None
	Fully connected layer	1 × 256 × 242	1 × 1 × 242	LReLu/None
Concatenate	Concatenate: clinical (14) and radiomic (242) features		1 × 1 × 256	None
Hidden layer: Predictor	Fully connected layer	1 × 242 × 256	1 × 1 × 256	LReLu/None
	Fully connected layer	1 × 256 × 128	1 × 1 × 128	LReLu/None
	Fully connected layer	1 × 128 × 64	1 × 1 × 64	LReLu/None
	Fully connected layer	1 × 64 × 32	1 × 1 × 32	None/None
Output layer	Fully connected layer	1 × 32 × 1	1 × 1	

^†^ A total of 36 axial images from the four different image series were incorporated into our model. * Leaky rectified linear unit (LReLu) activation function.

## Supplementary Materials

The following are available online at https://www.mdpi.com/2072-6694/12/8/2284/s1, Figure S1: The automatically determined weights for generating a weighted image to achieve high prediction accuracy for OS.
