# Supplementary material for: Multi-Parametric Deep Learning Model for Prediction of Overall Survival after Postoperative Concurrent Chemoradiotherapy in Glioblastoma Patients
Source: Cancers (Basel). 2020 Aug 14;12(8):2284. doi: 10.3390/cancers12082284 (PMC7465791; doi:10.3390/cancers12082284)
Supplement: Supplementary file 1 [file cancers-12-02284-s001.pdf]

Supplementary Material: Multi-Parametric Deep Learning Model for Prediction of Overall Survival after Postoperative Concurrent Chemoradiotherapy in Glioblastoma Patients

Han Gyul Yoon, Wonjoong Cheon, Sang Woon Jeong, Hye Seung Kim, Kyunga Kim, Heerim Nam, Youngyih Han and Do Hoon Lim

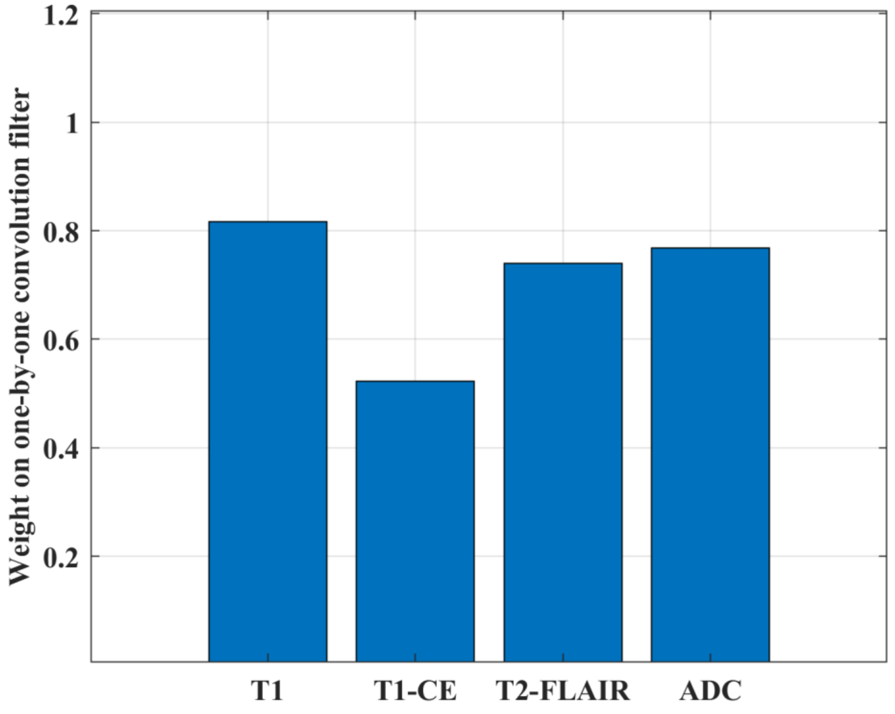

Figure 1. The automatically determined weights for generating a weighted image to achieve high prediction accuracy for OS.
